# Experimental risk assessment for chikungunya virus transmission based on vector competence, distribution and temperature suitability in Europe, 2018

**DOI:** 10.2807/1560-7917.ES.2018.23.29.1800033

**Published:** 2018-07-19

**Authors:** Anna Heitmann, Stephanie Jansen, Renke Lühken, Michelle Helms, Björn Pluskota, Norbert Becker, Carola Kuhn, Jonas Schmidt-Chanasit, Egbert Tannich

**Affiliations:** 1 Bernhard Nocht Institute for Tropical Medicine, Hamburg, Germany; 2 These authors contributed equally to this work; 3 German Centre for Infection Research (DZIF), partner site Hamburg-Luebeck-Borstel-Riems, Hamburg, Germany; 4 Institute for Dipterology (IfD), Speyer, Germany; 5 University of Heidelberg, Heidelberg, Germany; 6 German Environment Agency (UBA), Berlin, Germany

**Keywords:** chikungunya virus, *Aedes albopictus*, transmission rate, risk assessment, Europe

## Abstract

Over the last decade, the abundant distribution of the Asian tiger mosquito *Aedes albopictus* in southern Europe and the import of chikungunya virus (CHIKV) by infected travellers has resulted in at least five local outbreaks of chikungunya fever in France and Italy. Considering the ongoing spread of *Ae. albopictus* to central Europe, we performed an analysis of the Europe-wide spatial risk of CHIKV transmission under different temperature conditions. **Methods:** *Ae. albopictus* specimens from Germany and Italy were orally infected with CHIKV from an outbreak in France and kept for two weeks at 18 °C, 21 °C or 24 °C. A salivation assay was conducted to detect infectious CHIKV. **Results:** Analyses of mosquito saliva for infectious virus particles demonstrated transmission rates (TRs) of > 35%. Highest TRs of 50% for the mosquito population from Germany were detected at 18 °C, while the Italian population had highest TRs of 63% at 18 °C and 21 °C, respectively. Temperature data indicated a potential risk of CHIKV transmission for extended durations, i.e. sufficiently long time periods allowing extrinsic incubation of the virus. This was shown for areas already colonised by *Ae. albopictus*, as well as for large parts of central Europe that are not colonised. **Conclusion:** The current risk of CHIKV transmission in Europe is not primarily restricted by temperature, which allows extrinsic incubation of the virus, but rather by the vector distribution. Accordingly, all European countries with established populations of *Ae. albopictus* should implement respective entomological surveillance and monitoring systems, as basis for suitable control measures.

## Introduction

Chikungunya virus (CHIKV) is a mosquito-borne alphavirus (Togaviridae family) [1]. The virus was first isolated from humans, as well as from mosquitoes, during an outbreak at the border region between Mozambique and Tanzania in 1952. Human cases of chikungunya fever can cause severe, debilitating and often chronic arthralgia [2]. Several species of the mosquito genus *Aedes* were found competent to transmit CHIKV. However, *Ae. aegypti* and *Ae. albopictus* are considered the most important vectors. In the past, occurrences of CHIKV were restricted to the African and Asian continents, causing local, sporadic epidemics; during the last decade, however, the virus has expanded over a substantial geographical range, invading India, Indian Ocean islands and the Americas, where it has caused millions of human infections [2].

Three phylogenetic CHIKV lineages with distinct antigenic characteristics are known worldwide [2,3]. Two major lineages circulate in Africa (East, Central and Southern Africa region (ECSA) and West Africa) and a third is present in Asia. In Africa, CHIKV is maintained in an enzootic cycle. The transmission cycle includes forest-dwelling mosquito species of the genus *Aedes* and non-human primates [2]. Anthropophilic mosquito species, *Ae. aegypti* and *Ae. albopictus*, play a crucial role in the urbanisation of CHIKV. This is in contrast to Asia, where the virus has historically been maintained in an urban cycle between *Ae.* *aegypti*/*albopictus* and humans.

*Ae. aegypti* is the primary vector for CHIKV. However, *Ae. albopictus* is considered to have a high competence to transmit a specific variant of the ECSA lineage. The virus mutant has a single amino acid change from alanine to valine at the E1 envelope glycoprotein amino acid 226 (E1–A226V) [4]. This change leads to a better adaptation of the virus to the species *Ae. albopictus*, resulting in a 50-fold increase in vector competence in comparison to *Ae.* *aegypti*. The ECSA mutant is considered to be the most important factor for CHIKV outbreaks in regions where the primary vector, *Ae.* *aegypti*, is absent. This is potentially linked to the spread of the ECSA lineage to the Indian Ocean and the first autochthonous CHIKV transmission in Europe [5]. Thus far, all CHIKV strains isolated during outbreaks in Europe belonged to the ECSA lineage. However, some of the isolates contained the E1-A226V-mutation [6-8], whereas others did not [9,10]. Therefore, other yet undefined mutations in the envelope proteins may also affect the adaptation of CHIKV to *Ae. albopictus* [9-11].

Autochthonous transmission of CHIKV has been repeatedly observed in mainland Europe. The virus circulated in France in 2010 [9], 2014 [7] and 2017 [8], and two major outbreaks occurred in Italy in 2007 [6] and 2017 [10]. In total, at least 605 human CHIKV cases were reported, the majority in the two epidemics in Italy (n = 575) [8]. This was possible because of the establishment of *Ae. albopictus* in the region. One of the most invasive mosquito species in the world [12,13], its global spread is driven by transcontinental connectivity through shipping and flight routes. At present, *Ae. albopictus* has infested more than 25 European countries [12,14], with highest abundances reported from Italy. The species is repeatedly introduced to various locations in central Europe [15], even though regions north of the Alps were previously considered unsuitable for the establishment of *Ae. albopictus*. Still, overwintering was recently suggested in Germany [16-18]. This included local expansion of populations and detection of larvae already in spring. According to the European Centre for Disease Prevention and Control (ECDC), Solna, Sweden, *Ae. albopictus* is classified as ‘established’ along the Upper Rhine Valley in Germany and France [14].

There is currently no available vaccine or CHIKV-specific treatment [5]. The control of the disease primarily depends on reduction of the vector population. Further options are individual protection with repellents or behavioural avoidance. Therefore, the evaluation of the vector competence of local mosquito populations is crucial, as the assessment of CHIKV risk transmission allows for the adapting of surveillance and control systems. Vector competence is the ability of a mosquito to acquire a pathogen and subsequently transmit it to a new host [2]. This is commonly evaluated through experimental infection experiments. These studies aim to identify infectious virus particles secreted with the saliva to the vertebrate host. Transmission only becomes possible if the virus can overcome the midgut and salivary glands, which are the most important barriers for infection of the vector and final escape [19]. Vector competence depends on a complex interaction of vector population, virus strain and temperature [20]. Only a suitable combination allows the virus to replicate and disseminate. Invasion of the salivary glands may result in transmission through the next bite.

Vector competence studies using temperatures below 20 °C have become increasingly important since the observation of established populations of the *Ae. albopictus* north of the Alps [14]. We therefore conducted a vector competence study at three different temperatures, with a CHIKV outbreak strain from France, using *Ae. albopictus* populations from Germany and Italy, to comprehensively assess the risk of CHIKV transmission in Europe.

## Methods

### Source, rearing and experimental infection of *Aedes albopictus*

*Ae. albopictus* populations were obtained through the collection of eggs during August 2015. Samples originated from a long-standing population in Italy (Mandatoriccio, Calabria) and a recently established population in Germany (Freiburg, Baden-Wuerttemberg). Colonies were reared at 26 °C with a relative humidity of 80% and a 12:12 hour light:dark photoperiod. Mosquitoes of generation F8 to F10 were used for experiments. Between 100 and 150 female mosquitoes (4–10 days old) were sorted in Drosophila breeding vials (Carl Roth, Karlsruhe; 20 mosquitoes each). All specimens were starved for 24 hours before they were fed with artificial, infectious blood meals. Blood meals contained 50% of human blood from the blood bank, 30% fructose at a stock concentration of 8%, 10% filtrated bovine serum (FBS) and 10% of CHIKV stock (strain CNR_24/2014, supplied by the European Virus Archive goes Global project, ECSA lineage, isolated from a human case in France, grown on Vero cells, fifth passage). Blood meals had a final concentration of 10^6^ plaque-forming units of CHIKV per mL. Two droplets of 50 µl per vial were offered for 2 hours. Engorged mosquito specimens were sorted in new Drosophila breeding vials. Groups of 10 to 20 individuals were incubated at either 18 °C, 21 °C or 24 °C with 80% humidity for 14 days. These temperature conditions represent different areas in Europe infested by *Ae. albopictus*.

The central European areas recently invaded by *Ae. albopictus* only reach mean daily temperatures of ≥ 18 °C or ≥ 21 °C for 2 weeks per year. Areas with long-standing populations around the Mediterranean Sea can also have 14 days with temperatures ≥ 24 °C.

### Collection of infection and transmission rates

Subsequently, mosquitoes were analysed for virus infection and transmission. Infection of mosquito bodies was determined by analyses of head, thorax and abdomen. Legs and wings were removed for demobilisation in the salivation assay. Each body was homogenised in 500 µl of Dulbecco’s Modified Eagle Medium (Sigma-Aldrich, St. Louis, Missouri, United States (US)). Viral RNA was extracted using the 5x MagMax Pathogen RNA/DNA Kit (Thermo Fisher Scientific, Waltham, Massachusetts, US). Detection of CHIKV RNA was conducted with RT-PCR (RealStar Chikungunya RT-PCR Kit 2.0, altona Diagnostics, Hamburg, Germany). The detailed salivation assay to detect transmission was described before [21]. In brief, mosquitoes were anesthetised with CO_2_ and demobilised. Forced salivation was conducted by placing the proboscis of the mosquito into a filter tip filled with 10 µl of PBS for 30 min. Saliva was incubated on Vero cells for 4 days and cells were checked for cytopathic effect (CPE). The supernatant of CPE-positive cells was harvested. RNA copies were detected using the QIAamp Viral RNA Mini Kit (Qiagen, Hilden, Germany) and the RealStar Chikungunya RT-PCR Kit 2.0.

Infection rate (IR) is commonly defined as the number of CHIKV-positive mosquito bodies per number of fed females. Different definitions exist for the calculation of transmission rates (TRs). The study did not focus on the mechanistic processes of vector competence (e.g. infection barriers), but aimed to assess the risk for CHIKV transmission in Europe. Therefore, in order to simplify the sample processing, the calculation of TRs followed the definition by Fortuna et al. [22]: number of mosquitoes with CHIKV-positive saliva per number of mosquitoes with CHIKV-positive bodies.

### Chikungunya virus transmission risk assessment

The risk map for CHIKV transmission in Europe was constructed by combining the current distribution of *Ae. albopictus* with temperature data from the infested regions. Current distribution data of *Ae. albopictus* at the regional administrative level (NUTS3), as at April 2018, were obtained from [14]. Time series of daily mean temperature data (European re-analysis and observations for monitoring, E-OBS v17.0) for the study area were downloaded from http://www.ecad.eu [23]. E-OBS data are available on a 0.25 ° regular latitude-longitude grid and were extracted for a period of 10 years between 2008–2017. For each grid cell, the number of days per year with preceding 14 days having a mean daily temperature ≥ 18 °C were calculated using the programme R [24]. The annual values were then averaged over the 10-year period.

## Results

Experimental infection studies showed IRs of 100%, irrespective of whether *Ae. albopictus* populations from Germany or Italy were used and independent of temperature (18 °C, 21 °C or 24 °C) (Table). Virus titres for both populations were similar. The values ranged from 10^8.4^ to 10^11.3^ viral RNA copies per specimen, but were substantially higher at 18 °C compared with 24 °C for both populations. Likewise, infectious CHIKV particles were detected in mosquito saliva for all of these combinations, with TRs between 37.5% and 63.3%. Lowest TRs (37.5%) for both populations were observed at the warmest temperature tested (24 °C). In contrast, the highest TR (50%) for the population from Germany was detected at the coolest temperature (18 °C). The population from Italy had similar high TRs of 63.3% at 18 °C and 21 °C, respectively.

**Table:** Infection rates, transmission rates and virus titres of *Aedes albopictus* specimens from two different European populations experimentally infected with chikungunya virus and kept at three different temperatures, November 2017–March 2018 (n = 163 mosquitoes)

*Aedes albopictus* population origin	T in °C	Infection rate^a^		Transmission rate^b^		Titre	
		Number of CHIKV-positive specimens/number of analysed specimens	%	Number of specimens with CHIKV-positive saliva/number of specimens with CHIKV-positive body	%	Mean log^10^ CHIKV RNA copies/specimen	SD
Germany	18	32/32	100.0	16/32	50.0	11.3	0.6
Germany	21	23/23	100.0	9/23	39.1	8.4	0.8
Germany	24	24/24	100.0	9/24	37.5	8.5	0.2
Italy	18	30/30	100.0	19/30	63.3	10.8	1.8
Italy	21	30/30	100.0	19/30	63.3	10.4	1.6
Italy	24	24/24	100.0	9/24	37.5	8.6	0.2

CHIKV: chikungunya virus; SD: standard deviation; T: temperature.

^a ^Infection rate: number of CHIKV-positive mosquito bodies per number of fed females.

^b ^Transmission rate: number of mosquitoes with CHIKV-positive saliva per number of mosquitoes with CHIKV-positive bodies.

### Chikungunya virus transmission risk assessment

The analysed data indicated that most parts of the vector’s current distribution area allow transmission of CHIKV (Figure). All of these areas have sufficiently long time periods with mean daily temperatures ≥ 18 °C. The only exceptions were regions within the Alps and the Pyrenees; these either have very low vector densities or represent data artefacts, i.e. belonged to a NUTS3 unit infested by *Ae. albopictus* at lower altitudes. In addition, more parts of central Europe (e.g. Belgium, Poland) have suitable temperature conditions, but are not colonised by *Ae. albopictus*. The largest time windows with temperatures ≥ 18 °C are found close to the Mediterranean Sea. For example, the coastline of France and large parts of Italy have more than 120 days per year with temperatures that could allow CHIKV transmission. In addition, the temperatures in the Upper Rhine Valley, the northernmost region with established *Ae. albopictus* populations, theoretically allow extrinsic virus incubation. These areas in Germany and France have periods of more than 40 days per year, on average, that are suitable for CHIKV transmission.

**Figure:**
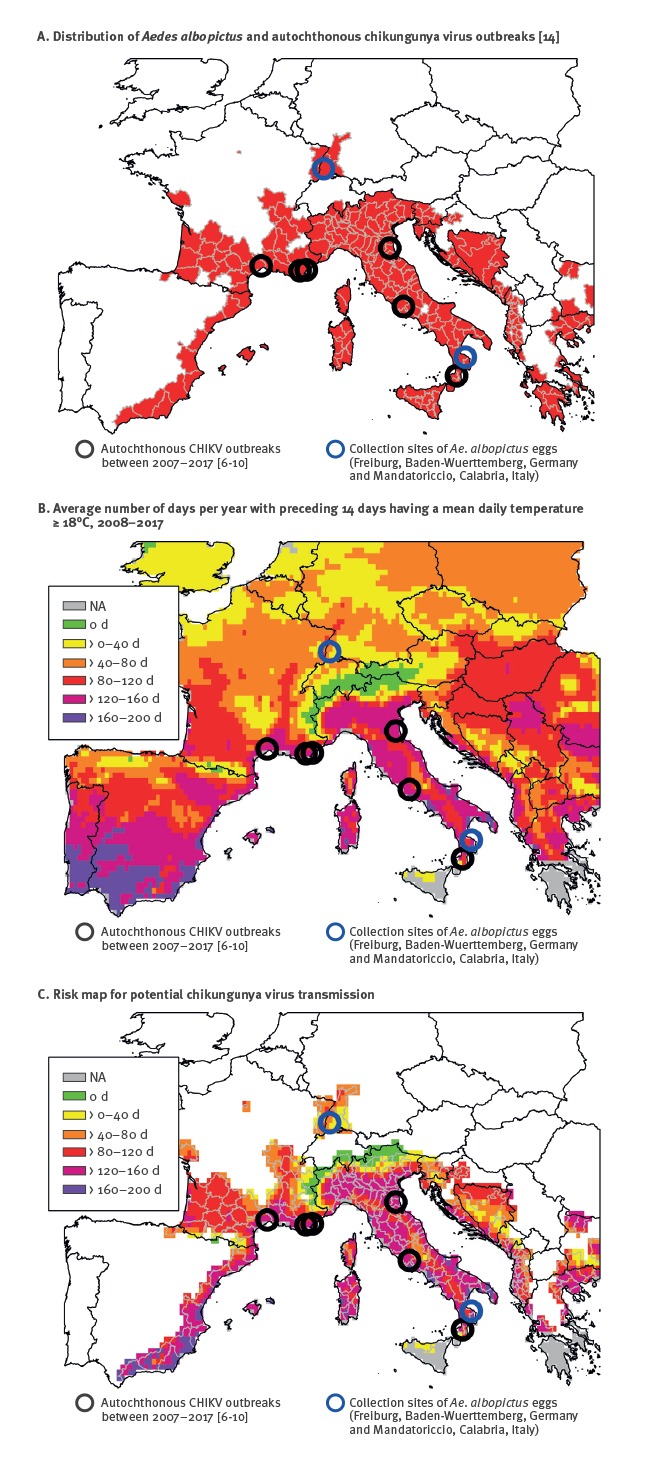
(A) Distribution of *Aedes albopictus *[14], (B) average number of days per year with preceding 14 days having a mean daily temperature ≥ 18 °C, 2008–2017 and (C) risk map for potential chikungunya virus transmission, Europe, 2018

CHIKV: chikungunya virus; d: days; NA: no temperature data available.

## Discussion

The extrinsic incubation period (EIP) of CHIKV in *Ae. albopictus* can be very short, i.e. infectious particles can be present in the saliva within 2 to 3 days after ingestion of an infectious blood meal [25]. This has a direct impact on the epidemiology of CHIKV. It must be considered one of the most important factors allowing for transmission in areas without tropical climates. However, there is a lack of comprehensive knowledge regarding the EIP of CHIKV, especially at low temperatures [26]. Only a few studies have focused on temperate climatic conditions [2]. Such studies are especially important in light of the ongoing spread of *Ae. albopictus* from source populations around the Mediterranean Sea to central Europe [14-18].

The tested Italian mosquito population, as well as the more recently detected German mosquito population, showed IRs for CHIKV of 100%. TRs of more than 35% were observed for all three tested temperatures. It may appear surprising that the lowest CHIKV TRs (37.5%) for both populations were found at the highest temperature (24 °C) and TRs increased with decreasing temperatures; however, this is in line with previous infection studies with different arboviruses [27]. Some mosquito species have a reduced ability to modulate viral infections under low temperatures. The underlying mechanism might be a temperature-dependent deficiency of antiviral immunity, as RNA silencing is inhibited in mosquitoes subjected to low temperatures. *Ae. aegypti* specimens reared at cooler temperatures have an impairment of the antiviral immune RNA interference (RNAi) pathway. This pathway is critical to the mosquito’s ability to control viral infections. The exact mechanism between temperature and CHIKV IRs needs to be further explored, even though it is well established that RNAi impairments occur downstream of the initial dicing step [27].

Human CHIKV infections are regularly imported to Europe [28]. The two large outbreaks in Italy also affected visitors (including a German traveller in 2017; personal communication, Christina Frank, Robert Koch-Institute, Germany, November 2017).* Nevertheless, the current risk of CHIKV transmission to humans is predominantly restricted by the presence of *Ae. albopictus*. Local temperatures allowing extrinsic incubation of the virus only play a minor role. Major parts of the currently infested European areas could allow CHIKV transmission. These areas have periods with 14 consecutive days of mean daily temperatures ≥ 18 °C. This also applies to the Upper Rhine Valley in Germany and France, where recently established populations were documented. Temperature data also illustrate a potential risk for areas in central Europe that are not colonised by *Ae. albopictus*. This underlines the importance of controlling any further spread of *Ae. albopictus* in Europe.

Three outbreaks in France and two in Italy between 2007–2017 highlight the risk of autochthonous CHIKV transmission in Europe [6-10]. Notably, these sites were all characterised by time periods of over 120 days allowing CHIKV transmission per year. Results of the study presented here are in line with previous investigations. European populations of *Ae. albopictus* have vector competence for CHIKV at temperate temperature conditions [5]. In contrast, recent modelling studies identified only a moderate risk for CHIKV transmission in Europe [26,29]. However, as discussed by Tjaden et al. [26], these studies are probably biased by the sparse number of CHIKV records for the region.

The experiments presented in this study used two different European populations of *Ae. albopictus* at three different temperatures, but only one CHIKV strain. Different CHIKV strains may differ in their potential to be transmitted by *Ae. albopictus*. The presented infection experiments were conducted with the ECSA strain (CNR_24/2014) that originates from the 2014 CHIKV outbreak in France and carries the E1–A226V mutation, which enhances the vector competence of *Ae. albopictus*. This might be one explanation for the observation of high IRs and TRs for both *Ae. albopictus* populations at the various temperatures studied. However, at least two European CHIKV outbreaks in France (2010) and in Italy (2017) were caused by virus strains without this specific mutation [8,10]. Further mutations might have a similar relevance for the probability of transmission in Europe [9,10]. Potential candidates are adaptive mutations in the E2 envelope glycoprotein (e.g. E2-L210Q) [11]. Such mutants were detected in CHIKV isolates from India and might play an important role in the spread and diversification of CHIKV lineages. In addition, one must keep in mind that vector competence is an important parameter of vector capacity, but it is not the only one. Vector capacity is determined by a complex interaction between different factors [30]. Important drivers include the population density and host-feeding patterns. The latter is strongly influenced by both host preference and host availability.

The risk assessment presented here is a conservative scenario. Both tested populations had substantial CHIKV TRs at 18 °C, i.e. 14 days post infection. In addition, temperature data for the risk maps were averaged over a 10-year period. For a more comprehensive and precise evaluation, further vector competence studies investigating lower temperatures and shorter EIPs are required. In addition, this analysis has only focused on *Ae. albopictus*, though other studies have demonstrated vector competence for CHIKV of further *Aedes* species [2]. Infection experiments have not identified any other European mosquito species except *Ae. albopictus* as potential vector for CHIKV, but only a few studies have been done for selected species (e.g. *Ae. vexans* and *Culex pipiens*).

In conclusion, Europe offers broad temperature suitability for CHIKV transmission and experiences regular travel-associated virus introduction. Therefore, all European countries with established *Ae. albopictus* populations should implement entomological surveillance programs as well as monitoring and notification systems for imported human cases to prevent further spread and autochthonous CHIKV transmission in Europe.

## *Authors’ correction

In the Discussion, the sentence “Further spread of the virus was observed during the two large outbreaks in Italy (e.g. to Germany; personal communication, Christina Frank, Robert Koch-Institute, Germany, November 2017)." was replaced by "The two large outbreaks in Italy also affected visitors (including a German traveller in 2017; personal communication, Christina Frank, Robert Koch-Institute, Germany, November 2017)." to avoid possible misinterpretation. The correction was made on 20 July 2018, as requested by the authors.
