# Authors’ correction for Euro Surveill. 2018;23(29)

**DOI:** 10.2807/1560-7917.ES.2018.23.30.1807264

**Published:** 2018-07-26

**Authors:** Eurosurveillance editorial team

**Affiliations:** 1 European Centre for Disease Prevention and Control (ECDC), Stockholm, Sweden

As requested by the authors of the article ‘Experimental risk assessment for chikungunya virus transmission based on vector competence, distribution and temperature suitability in Europe, 2018’, published on 19 July 2018, the sentence ‘Further spread of the virus was observed during the two large outbreaks in Italy (e.g. to Germany; personal communication, Christina Frank, Robert Koch-Institute, Germany, November 2017).’ was replaced by ‘The two large outbreaks in Italy also affected visitors (including a German traveller in 2017; personal communication, Christina Frank, Robert Koch-Institute, Germany, November 2017).’ to avoid possible misinterpretation. The correction was made on 20 July 2018.

